# Emergency repair of traumatic avulsion of the right main stem bronchus using biliary stent in a 6-year-Old girl: A case report

**DOI:** 10.1016/j.amsu.2022.104973

**Published:** 2022-11-17

**Authors:** Salem M. Tos, Afnan W.M. Jobran, Narmeen Giacaman, Mohammad G. Ibdah, Anas Alasafrah, Isam Shammas, Hazem Al Ashhab, Yousef Abu Asbeh

**Affiliations:** a Faculty of Medicine, Al Quds University, Jerusalem, Israel; b Al Ahli Hospital, Hebron, Palestine; c Chief of Surgical ICU Department, Al Ahli Hospital, Hebron, Palestine; d Al-Quds University Medical School, Palestine; e Thoracic Surgery Unit, Al Ahli Hospital, Hebron, Palestine

## Abstract

**Introduction:**

Pediatric thoracic trauma is a rare condition, but results in high levels of morbidity and mortality. These injuries are often more devastating in the pediatric population due to differences in children's anatomy and physiology relative to adult patients. Tracheobronchial injuries secondary to blunt trauma are difficult to diagnose compared to penetrating trauma. So, a high index of suspicion is of utmost importance.

**Case presentation:**

We report a case of a 6-year-old girl who experienced complete avulsion of the right main bronchus. The patient presented with extensive emphysema and severe respiratory distress with bilateral tension pneumothorax. Endotracheal intubation and two thoracostomy tubes were applied. Pneumothorax and a massive air leak persisted on the right side. After thoracotomy, right main bronchus avulsion was present and then repaired by end-to-end anastomosis and muscle flap. minimal air leaks from right chest tubes and partially expanding right lung on chest X-ray are seen after that. So, reinforcement by biliary (instead of bronchial) stent was performed because unavailability of bronchial stent with the desired size at that time.

**Discussion:**

Traumatic tracheobronchial damage occurs in just 0.05–3% of all pediatric thoracic traumas. It's fatal condition especially in first hour. With proper recognition and management of these injuries, there is an associated improved long-term outcome. This article reviews the current literature and discusses the initial evaluation, current management practices, and future directions in pediatric thoracic trauma.

**Conclusion:**

Biliary stent placement could be a reasonable treatment option for tracheobronchial damage.

## Highlights

• Main stem bronchus avulsion is a rare but fatal condition, especially in the first hour.
• Careful history taking, meticulous physical examination, and investigations are necessary.
• Prompt treatment should be address in a proper way to stabilize the patient and avoid mortality.

## 1 Introduction

Thoracic trauma is a rare condition, which present in only 13% of pediatric trauma patients. More than 80% of the affected patients suffer from severe multiple injuries after road traffic injuries or a fall [1]. Traumatic lung contusion is the most common type of blunt chest trauma in the pediatric population [2].

The force of the chest trauma and the resulting contusion can be massive without causing a fracture due the more pliable cartilaginous rib cage in children. Traumatic rupture and avulsion of the tracheobronchial tree requires even greater force. And therefore it is considered very rare injury, especially in pediatric population. Of these injuries, the most common site is Right main bronchus [3,4]. Traumatic bronchial rupture and avulsion often fatal, with mortality up to 30%, with one half of the deaths occurring during the first hour [5]. Tracheobronchial injuries secondary to blunt trauma are difficult to diagnose, compared to tracheobronchial injuries secondary to penetrating trauma which are easily identified [6].

In most cases of lower airway injuries, a simple bronchial repair by primary anastomosis is successful [7,8]. Coverage of the bronchial stump with intercostal flap is also possible [9], especially if primary anastomosis failed. Here, we present the initial airway management and surgical approach of a complete blunt right main bronchus avulsion, which was treated successfully using biliary (instead of bronchial) stent to reinforce muscle flap around primary anastomosis.

This work has been reported in line with the SCARE criteria, which is used by authors, journal editors and reviewers to increases the robustness and transparency in reporting surgical cases [10].

## 2 Case presentation

A 6-year-old girl presented to the Emergency Department after an accidental fall from a height of 4 m in a state of unconsciousness by an ambulance. She was found with a significantly decreased peripheral O_2_ saturation (SpO_2_) of 50% after application of supplemental oxygen, pulse 140 beats/minute; blood pressure was 80/50 mm Hg, a body temperature of 37.8 °C. Glasgow Coma Score (GCS) was 5/15. On physical examination, there were bruises on right upper chest with massive subcutaneous emphysema of the abdomen, chest, neck and face. The abdomen was distended. The breath sounds decreased on both sides equally, so bilateral needle thoracostomy was performed, and the patient was endotracheally intubated. After intubation and positive-pressure ventilation, oxygen saturation and blood pressure dropped more. At this time, bilateral tension pneumothorax was diagnosed, and two 20-mm thoracostomy tubes were placed in the ER. Then the patient transferred to Surgical Intensive Care Unit (SICU).

### 2.1 Diagnostic assessment

**Fig. 1:**
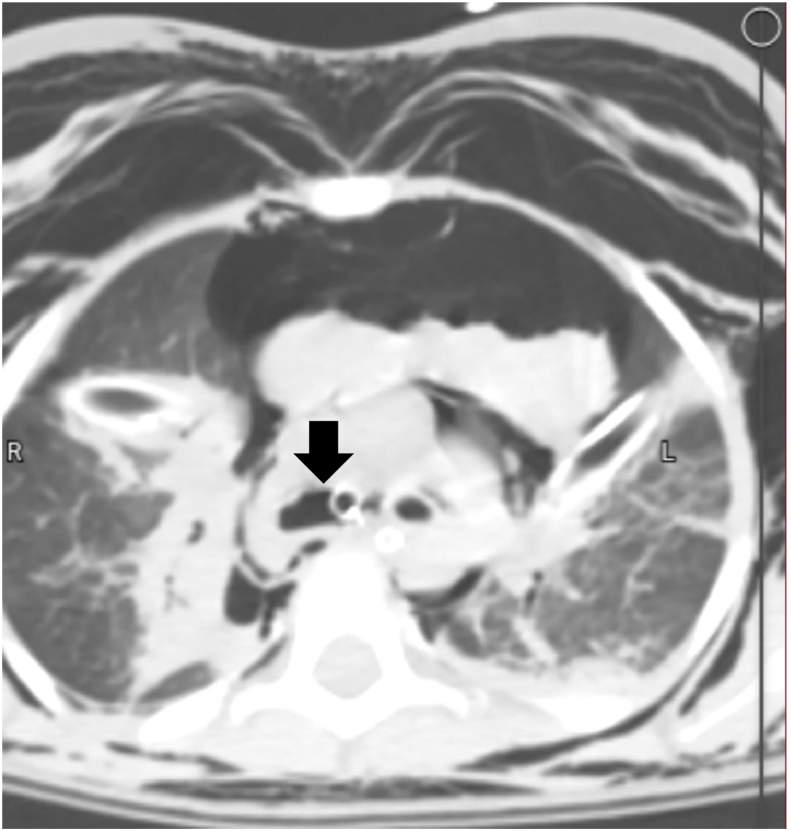
Axial view of thoracic CT scan (lung window) at the carina level, which demonstrates right main bronchus avulsion (arrow).

A massive air leak was appreciated from the chest tubes. The SaO_2_ improved to 80%. Computed tomography (CT) scan revealed a complete avulsion of the right main stem bronchus at the level of the carina (Fig. 1), a residual pneumothorax, large hematoma measured 3 x 8 × 9 cm in the anterior-middle mediastinum, atelectasis of the right lung, as well as emphysema of the mediastinum and soft tissue. The CT scan also showed few lacerations of the liver (largest one measured 1 × 2 cm) and spleen (less than 1cm in depth). It also showed Right adrenal hematoma measured about 3 × 2 cm and large wedge shaped Non-enhancing hypo-density of more than 50% of the right kidney suggesting kidney infarction. Moderate amount of abdomen-pelvic free fluid was also noted. The blood gas showed acidosis (pH 7.20), and hypercapnia (partial pressure of carbon dioxide 65 mm Hg).The patient was taken to the operating room immediately. With positive-pressure ventilation, oxygen saturation dropped continuously in spite of two working thoracostomy tubes and manual high frequency, low tidal ventilation.

### 2.2 Intraoperative

Under general anesthesia, while the patient placed in the left lateral decubitus position, an immediate posterolateral thoracotomy was performed. Almost complete avulsion of the right main bronchus from the trachea and accompanied continuous air leak were present. The defect was identified at the level of the carina. and Temporary occlusion of the defect with the surgeons' finger allowed some ventilation of the left lung to gradually improve the oxygen saturation. A sutured end-to-end anastomosis was performed and then reinforced by muscle flap as follows: An intercostal flap was dissected and harvested from the inferior margin of the fourth rib, with attention paid to avoid an injury to the vascular structures. After transection, the flap was then sutured to the bronchial stump with an interrupted absorbable suture (PDS 4/0 Ethicon). The time required for harvesting the flap was very short, about 5–10 minutes. There was no tension on the anastomosis and no air leak at under-water testing. Two chest tubes were left in place postoperatively.

### 2.3 Postoperative course

**Fig. 2:**
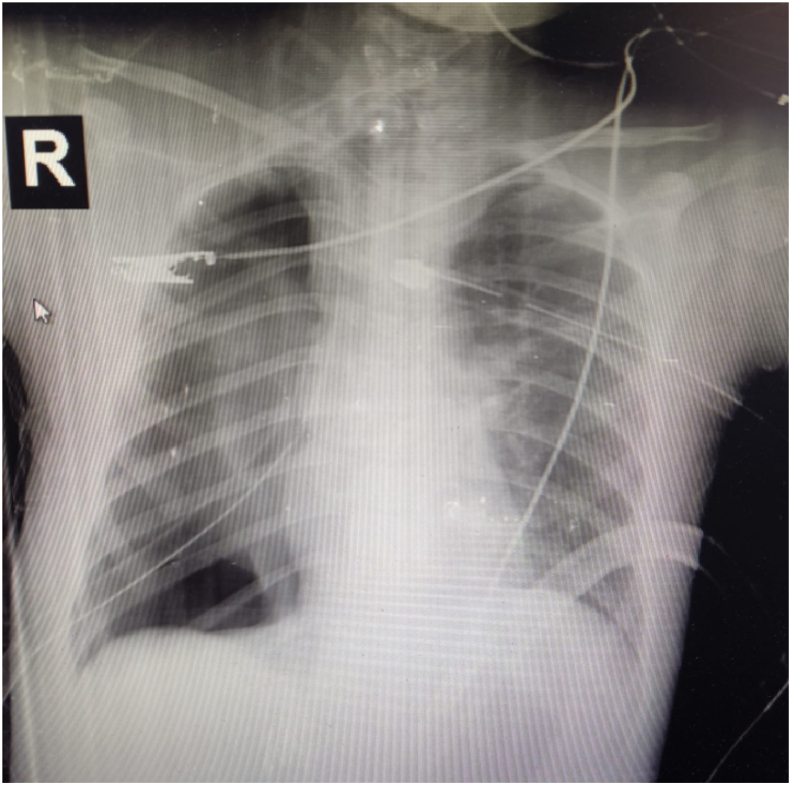
Supine radiograph shows a large right-sided pneumothorax after primary repair of right main bronchus avulsion despite use of chest drains.

The patient transferred back to SICU on mechanical ventilator, hemodynamically stable without support, but she was requiring respiratory support, pressure-controlled ventilation was continued, Broad-spectrum Antibiotics were administered. Serial Chest X-rays were performed, which showed fully expanded lung. Clinically, there were no air leaks and the patient's respiratory health was improved (but still on mechanical ventilator). However on the third postoperative day, air leaks was noticed in the chest tube, bronchoscopy was performed and showed the leak from muscle flap margin, and respiratory function deteriorate again. Second thoracostomy tube at right side was inserted, which showed continuous air leak. On the 5th post-operative day, tracheostomy (connected to mechanical ventilation) was applied. After 10 post-operative days, the patient improved clinically and mechanical ventilation was successfully discontinued. At this moment, while 2 right chest tubes in place, continuous air leaks still present (Figure-2). The patient was clinically stable, so we decided to continue on the conservative management after that.

**Fig. 3:**
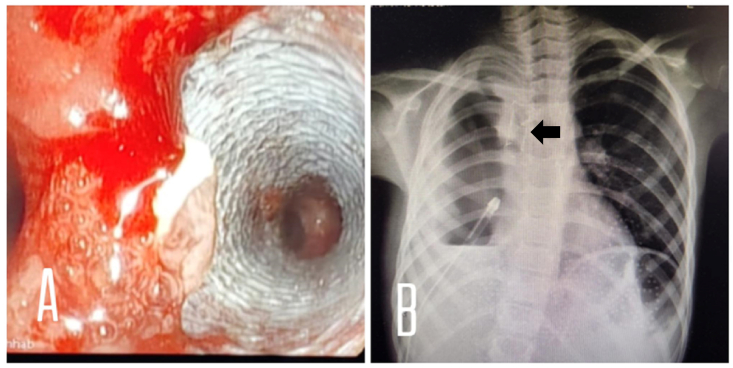
Bronchoscopic view (A) and chest X-ray (CXR) view (B) of a fully covered self-expandable metal stent (FC-SEMS) placed in right main bronchus (pointed by an arrow in CXR).

**Fig. 4:**
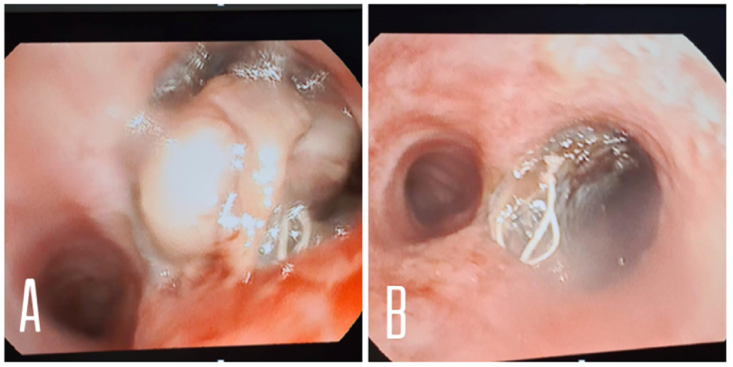
Bronchoscopic view revealed partieal obstruction in stent area due to thick lung secretions (A), removal of thick secretions and irrigation were performed after that (B). (Note: Both images were taken early as part of the serial daily bronchoscopies).

On day 20, the thoracic surgeon decided to reinforce the flap with a bronchial stent. Unfortunately, the bronchial stent with the desired size was unavailable in our hospitals yet. In consultation with both Gastroenterologist and General surgeon, there was a consensus to use biliary stent instead. Fully covered self-expandable metal stent (FC-SEMS) measured 10 mm × 50 mm was inserted by bronchoscopy (Fig. 3). And the patient was sent back to SICU under observation. After that, the air leak was absent and she improved clinically and the lungs were expanded partially. Serial chest X-rays were resumed. The patient underwent daily bronchoscopies for evaluation and to remove thick secretions and irrigation of the stent area (Fig. 4). On day 40 (20 days after placing the stent), due to residual space in the chest X-ray, we decided to proceed with VATS (video-assisted thoracic surgery) decortication of the right pleural space. The lungs were fully expanded post operative.

**Fig. 5:**
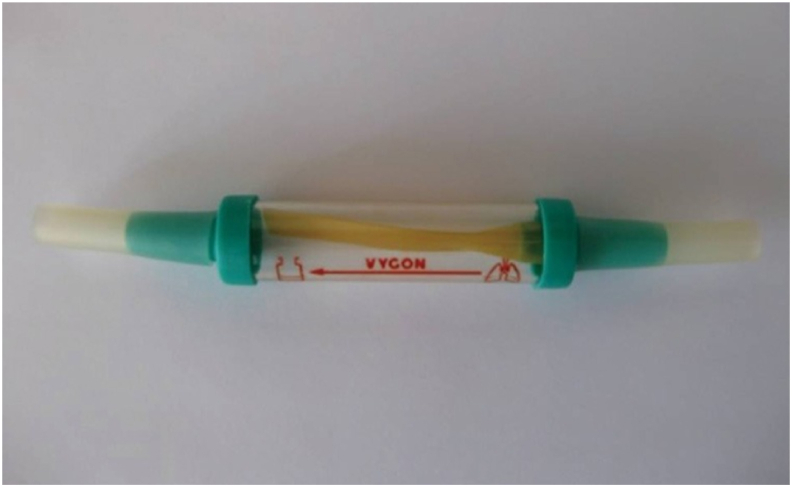
Heimlich flutter valve.

**Fig. 6:**
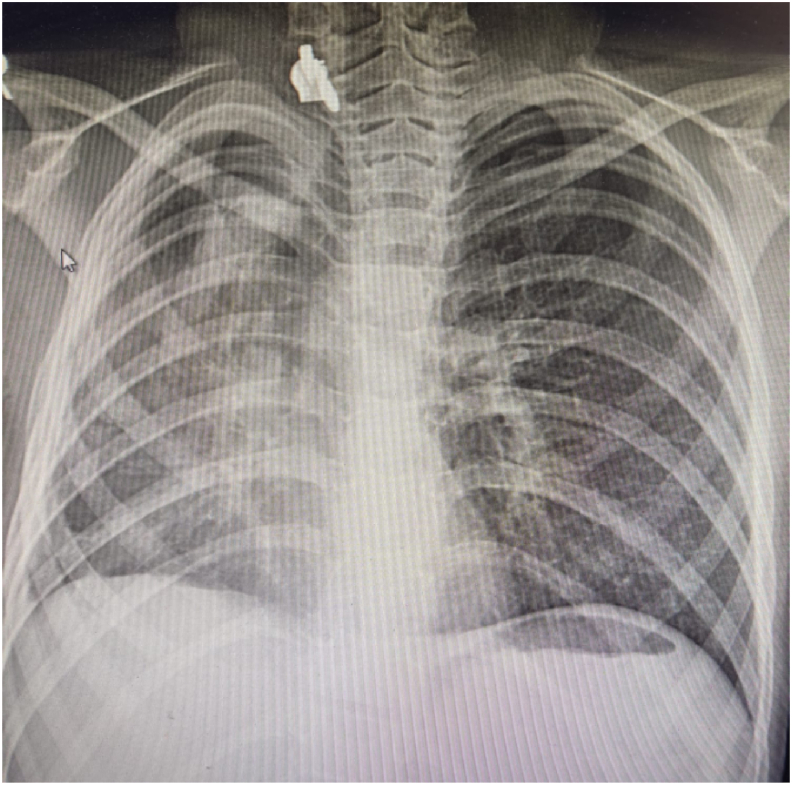
Chest radiography after stent removal which shows full expansion of the right lung.

The patient was discharged after 2 months of hospitalization with a Heimlich valve (Fig. 5), which was connected to the chest drainage tubes at Right chest wall. One month later, air leaking completely absent and both lung were fully expanding (Fig. 6). Heimlich valve, Chest tubes, and biliary stent were successfully removed postoperative Day 30. The patient had good general and respiratory health after that.

## 3 Discussion

Only 13% of pediatric trauma patients get thoracic trauma, making it a rare condition. More than 80% of the affected patients have serious combination injuries as a result of falls or injuries sustained in auto accidents [1]. The most frequent findings in pediatric patients are pulmonary contusions or rib fractures (50%) and pneumothorax (37%) [1]. Traumatic tracheobronchial damage, on the other hand, occurs in just 0.05–3% of instances [1]. These injuries are challenging to identify since their 2 clinical indicators might vary or be deceptive. Up to 30% of people who experience traumatic airway disruption die, and half of these deaths happen within the first hour after the event [8,11].

Children's injury patterns following blunt thoracic trauma are very different from those in adults. Children's thoracic walls have a high degree of flexibility, which allows external forces to affect the intrathoracic and mediastinal organs with little visible outward signs of injury [1,11]. Only 25% of tracheobronchial injuries, for instance, result in associated rib fractures. 2 Additionally, because of the mediastinum's greater mobility in children compared to adults, tension pneumothorax occurs three times more frequently in youngsters [1]. The earliest clinical symptoms of bronchial damage, such as dyspnea or subcutaneous emphysema, are non-specific, making the diagnosis challenging and potentially delaying treatment. In addition, several serious injuries are invariably caused by the high impact trauma that causes bronchial rupture.

Before inserting a chest tube, the patient was intubated, which resulted in a potentially fatal decompensation. Positive-pressure breathing should generally be avoided in patients with suspected bronchial damage since it could worsen the pneumothorax. A second chest tube should be inserted if the decompression of a pneumothorax from a single chest tube is insufficient. It is extremely suspicious of relevant airway injury if there is a significant, continuous air leak and increasing subcutaneous emphysema. This collection of symptoms needs to be recognized by clinicians.

For pediatric patients with thoracic trauma, a plain chest radiograph is typically advised since it can identify important fractures, pneumothoraxes, and mediastinal diseases that require rapid attention [1]. Regarding the principal application of chest CT in this group, there is no agreement. Even while a CT scan may be more sensitive to diseases such a minor pneumothorax or lung contusion, these additional findings infrequently result in a change in care.

More invasive surgical operations, including as a tracheostomy, tracheoplasty, thoracotomy, bronchoplasty, and/or extracorporeal life support, are typically required for victims who don't respond to medicinal care (ECLS). To preserve the patency of the airways, TB stents can be utilized to offer intraluminal structural support. Since the 1990s, metallic stent insertion has been promoted as a desirable choice for children because it can improve quality of life while delivering fast, stable, and long-lasting TB lumen patency [12]. This less invasive approach than surgical adjustments may help some people. Stents do have complications and related technical issues because they are foreign bodies, especially in pediatric patients. Most reported pediatric studies have been small in size and rarely given in detail on the metallic stent placement, maintenance, retrieval, and long-term outcomes.

The primary surgical strategy for treating tracheobronchial injuries in both adults and children is a thoracotomy and primary anastomosis without additional interventions. The location and length of the rip determine the surgical method that is used. Cervical incision is the most effective treatment for cervical trachea injuries. The distal trachea and mainstem bronchi are well exposed following a right posterolateral thoracotomy in the fourth intercostal gap. Only isolated transverse abruptions of the left mainstem bronchus should be treated with a left thoracotomy. According to the literature, running and interrupted sutures are both employed [8]. Inadequate suture pitches, differences in bronchial diameter, excessive stress on the anastomosis, and poor visibility of the operating field are risk factors for anastomotic leakage. In cases of discrepancy in bronchial diameter, bronchial plication of the membrane component of the bronchus, suture holders, or intercostal muscle flap may be utilized to lessen the risk of complications in a stable patient [13].

## 4 Conclusion

Bronchial damage is indicated by a collection of clinical symptoms including subcutaneous emphysema, refractory pneumothorax, and air leak from the thoracostomy tube. If at all possible, endotracheal intubation in these situations not performed until insertion of thoracostomy tube. Placing a retraction suture throughout repair is a technique that aids in closing the defect while maintaining the remaining tracheobronchial lumen running at the same time to ensure the contralateral lung receives vital breathing. Muscle flap and bronchial stent to reinforce defect closure and maintain patency are also possible.

## Provenance and peer review

Not commissioned, externally peer reviewed.

## Ethical approval

The study is exempt from ethical approval in our institution.

## Sources of funding

No funding or grant support.

## Author contribution

Study concept or design: Yousef Abu Asbeh.

Writing the manuscript: Salem M. Tos, Afnan W.M. Jobran, Narmeen Giacaman, Mohammad G. Ibdah, Anas Alasafrah, Isam Shammas and Hazem Al Ashhab.

Review & editing the manuscript: Salem M. Tos, Afnan W.M. Jobran.

## Trial register number

Not applicable.

## Guarantor

Dr. Yousef Abu Asbeh.

## Consent

Written informed consent was obtained from the patient's parents for publication of this case report and accompanying images. A copy of the written consent is available for review by the Editor-in-Chief of this journal on request.

## Declaration of competing interest

There is no conflict of interest.
